# Regulation of Cell Delamination During Cortical Neurodevelopment and Implication for Brain Disorders

**DOI:** 10.3389/fnins.2022.824802

**Published:** 2022-02-23

**Authors:** Godwin Sokpor, Beate Brand-Saberi, Huu Phuc Nguyen, Tran Tuoc

**Affiliations:** ^1^Department of Human Genetics, Ruhr University Bochum, Bochum, Germany; ^2^Department of Anatomy and Molecular Embryology, Ruhr University Bochum, Bochum, Germany

**Keywords:** delamination, neurepithelium, cortical development, cell adhesion, cell polarity, transcription factors, epigenetic regulation, neurodevelopmental disorders

## Abstract

Cortical development is dependent on key processes that can influence apical progenitor cell division and progeny. Pivotal among such critical cellular processes is the intricate mechanism of cell delamination. This indispensable cell detachment process mainly entails the loss of apical anchorage, and subsequent migration of the mitotic derivatives of the highly polarized apical cortical progenitors. Such apical progenitor derivatives are responsible for the majority of cortical neurogenesis. Many factors, including transcriptional and epigenetic/chromatin regulators, are known to tightly control cell attachment and delamination tendency in the cortical neurepithelium. Activity of these molecular regulators principally coordinate morphogenetic cues to engender remodeling or disassembly of tethering cellular components and external cell adhesion molecules leading to exit of differentiating cells in the ventricular zone. Improper cell delamination is known to frequently impair progenitor cell fate commitment and neuronal migration, which can cause aberrant cortical cell number and organization known to be detrimental to the structure and function of the cerebral cortex. Indeed, some neurodevelopmental abnormalities, including Heterotopia, Schizophrenia, Hydrocephalus, Microcephaly, and Chudley-McCullough syndrome have been associated with cell attachment dysregulation in the developing mammalian cortex. This review sheds light on the concept of cell delamination, mechanistic (transcriptional and epigenetic regulation) nuances involved, and its importance for corticogenesis. Various neurodevelopmental disorders with defective (too much or too little) cell delamination as a notable etiological underpinning are also discussed.

## Introduction

**FIGURE 1:**
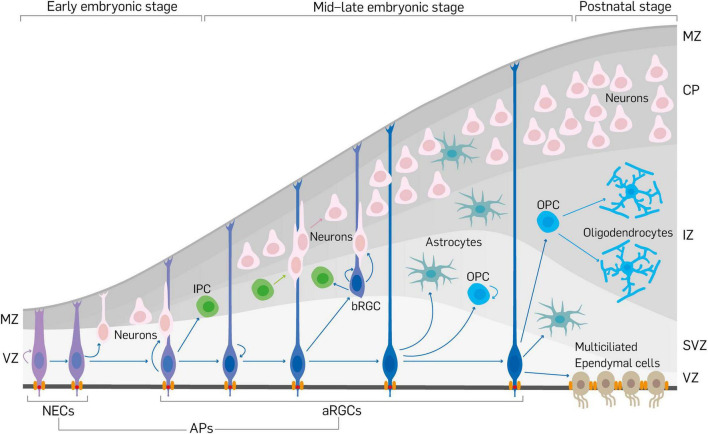
Schema showing neurodevelopment in the mouse cerebral cortex. Early in cortical development, neuroepithelial cells (NECs) first expand their pool via proliferation and then transform into apical radial glial cells (aRGCs). Together, NECs and aRGCs make the apical progenitors (APs) in the developing cortex. Apical progenitors are anchored at the ventricular surface by apical junctional complexes, and also establish basal connection beyond the marginal zone (MZ) to make them have apicobasal polarity. Apical progenitors can produce limited number of neurons by direct neurogenesis. The neuronal pool is increased by indirect neurogenesis via the generation of neuron-amplifying cells such as intermediate progenitor cells (IPCs) and basal radial glial cells (bRGCs) by aRGCs. Newborn neurons migrate by somal translocation or by locomotion using the slender fibers of aRGCs as guides to exit the ventricular zone/subventricular zone (VZ/SVZ) and through the intermediate zone (IZ) to reach their destination in the cortical plate (CP). In the course of corticogenesis, aRGCs give rise to or transform into glial cells (astrocytes, oligodendrocytes or oligodendrocyte precursor cells [OPCs], ependymal cells).

During early to mid-gestation morphogenesis of the cerebral cortex, many neurodevelopmental processes such as specification, proliferation, differentiation, and migration of neural cells (progenitors, neurons, and glia) are critically regulated to allow optimal formation of the neurons and glia. Fundamental among these events is the generation of primary neural progenitor cells known as apical progenitors (APs), which can transform into more differentiated cells or committed progenitors (Figure 1). APs are classically defined by their location in the ventricular zone (VZ), stemness/multipotency, and to a large extent their anchorage at the ventricular surface. As depicted in Figure 1, early in development, neuroepithelial cells mainly make up the AP population and later, following the onset of neurogenesis, they transition into apical radial glial cells (aRGCs) from which majority of the cortical parenchyma (neurons and glia) are derived (Götz and Huttner, 2005; Kriegstein and Alvarez-Buylla, 2009; Martínez-Cerdeño and Noctor, 2016). Although other types of APs, including short neural precursors (Gal et al., 2006), have been identified in the VZ, aRGCs are most abundant and principal in the generation of basal progenitors (BPs) that reside outside the VZ and more concentrated in the subventricular zone (SVZ) of the developing cortex (Figure 1). Intermediate progenitor cells (IPCs), and basal (outer) radial glial cells (bRGCs) are notable examples of BPs in the developing mammalian neocortex (Figure 1), with more prominence of the latter in the developing primate/human cortex (Haubensak et al., 2004; Miyata et al., 2004; Noctor et al., 2004; Kriegstein et al., 2006; Fietz et al., 2010; Hansen et al., 2010; Wang et al., 2011; Reillo and Borrell, 2012).

Some unique features of APs such as their integration in the adherens junction (AJ) belt, and proximity or exposure of part of their cell membrane to the lumen of the ventricle (Kriegstein and Götz, 2003; Götz and Huttner, 2005) are key determinants of their fate. The apicobasal polarity of APs allows them to undergo specific cellular dynamics in order to sustain their stem cell property and permits modulation of their proliferative and differentiative tendencies. During differentiative division of APs to neuronal or neurogenic cells, and perhaps transformation into glial cells (Figure 1), the pattern of inheritance of cell polarity, basal process, some cellular components, and the apical cell adhesion complex is a formidable predictor of the fate choice of daughter cells [reviewed in Kon et al. (2017)].

**FIGURE 2:**
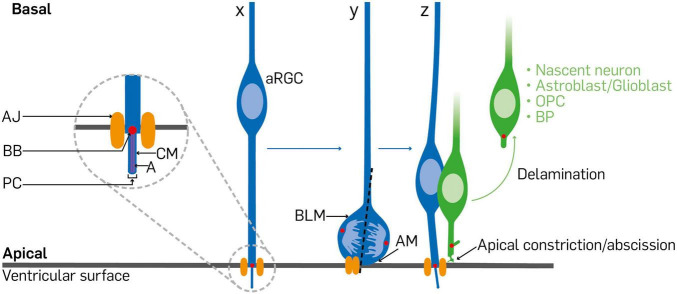
Diagram depicting synopsis of cell delamination in the cortical neurepithelium. A typical radial glial cell (x) is anchored at its endfoot by an apical junction/adherens junction. When the radial glial cell receives signals to undergo differentiative/asymmetric division, its soma is positioned at the ventricular surface. The inheritance or distribution of components of the dividing radial glial cell (y) is a determinant of fate of the progenies. The primary cilium and associated mother centrosome, apical membrane, and adherens junction are distributed among the daughter cells such that the one to maintain the radial glia identity bears the primary cilium and associated basal body, acquires most of the apical membrane, and reestablishes the adherens junction (z). The daughter cell poised to differentiate withdraws from the adherens junction belt via apical constriction or abscission at the ventricular surface as delaminated cell, which could end up as a basal progenitor cell, nascent neuron, or a glial cell/precursor that basally relocates in the cortical wall. aRGC, apical radial glial cell; AJ, adherens junction; BB, basal body; PC, primary cilium; CM, ciliary membrane; A, axoneme; BLM, basolateral membrane; AM, apical membrane; OPC, oligodendrocyte precursor cell; BP, basal progenitor.

A major mechanism by which APs differentiate to give rise to more committed progenies, such as BPs and neurons, is via the mechanism of cell delamination from the tethering AJ complexes and other restrictive apical elements in the VZ following mitotic cell division at the luminal surface (Figure 2; Haubensak et al., 2004; Miyata et al., 2004; Noctor et al., 2004; Fietz et al., 2010; Hansen et al., 2010; Das and Storey, 2014; Kasioulis et al., 2017; Camargo Ortega et al., 2019). The process of cell delamination has thus emerged as a pivotal neurodevelopmental event that is known to be delicately regulated to ensure proper cell number and location in the developing cortex. By determining delamination, such cell attachment regulatory factors prevent aberrant expansion of neural progenitor pool and untimely neuroglial differentiation. The said regulators of cell attachment, and for that matter delamination, can be categorized as transcriptional, chromatin remodeling and epigenetic factors, cell-cell interaction proteins, apical and junctional protein complexes, spindle positioning factors, and signaling modulators. This review succinctly discusses advances in our understanding of the molecular regulators of cell delamination as a critical cell biological event exhibited by apical neural stem/progenitor cells and their basal derivatives during corticogenesis. The consequence of dysregulated cell delamination is then placed within the context of pertinent neurodevelopmental disorders that affect normal brain structure and function.

## Key Morphological Features of Apical Progenitors Affected by Cell Attachment or Delamination Factors

The structural components in and around epithelial cells play key roles in determining their property and stability in the epithelium. Much as specific intracellular components of some cells can adapt them for unique functions or cause them to acquire other fates, some cellular structures capable of interacting with signals in the external cell environment can also influence cell fate or transition. This is particularly true for the cortical neuroepithelial cells, which possess structural features different from their progenies that adapt them for cellular processes, including cell delamination, known to underscore their proliferative and differentiative dispositions. The apical-basal (polarized) orientation of APs in the cortical wall is believed to establish differential receptive areas for developmental signals at either luminal (ventricular fluid) or adluminal (basal lamina) domains, and contribute to fate determination (Taverna et al., 2014). Some other morphological features of APs, particularly aRGCs, have been identified to be indispensable for their fate switch via cell delamination during cortical development. These are the apical plasma membrane, primary cilium and related centrosome, apical junctional complexes, and basolateral membrane. The description of these AP-related features and how they support cell delamination in the neurepithelium are discussed in the following subsections.

### Apical Plasma Membrane

The actual apical plasma membrane of an AP, which accounts for about 2% the entire plasma membrane and together with associated apical junctional belt, make the apical endfoot at the ventricular surface (Figure 2; Kosodo et al., 2004). This makes the apical plasma membrane to be in contact with the ventricular fluid, cerebrospinal fluid (CSF), to allow transductions of CSF-borne neurodevelopmental signaling molecules such as sonic hedgehog (Shh), fibroblast growth factors (FGFs), bone morphogenetic proteins (BMPs) insulin-like growth factors (IGFs), retinoic acid, Platelet Derived Growth Factors (PDGFs), and WNTs (McCarthy and Argraves, 2003; Lehtinen and Walsh, 2011; Segklia et al., 2012). It implies that there are several receptors localized at the apical domain that can bind to major signaling effectors belonging to the aforementioned signaling pathways capable of eliciting transcriptional and epigenetic responses in APs or neighboring cells. The area of apical membrane is known to progressively decrease as development advances. This has been attributed to the pinching off of extracellular membrane fragments (ectosomes) which contain some apical plasma membrane (Marzesco et al., 2005, 2009; Dubreuil et al., 2007). Apart from establishing polarity and receiving signals that influence brain developmental processes like patterning, cell migration and differentiation, the apical membrane may also determine the fate choice of AP progenies. During mitosis of AP cells, the inheritance of the apical membrane can be a decisive factor in cell division mode adoption (i.e., either proliferative symmetric or differentiative asymmetric division) and the fate specification of daughter cells (Kosodo et al., 2004). Of note, the apical plasma membrane usually bears another vital AP cell structure called the primary cilium which function together to establish the said polarity and a signaling hub that can drive cell fate transition (Louvi and Grove, 2011), and likely other developmental outcomes consequent to delamination.

### Primary Cilium

The primary cilium is a slender appendage of the apical plasma membrane that protrudes into the ventricular lumen and acts as an antenna for collecting developmental cues such as effector molecules in CSF for signal transduction (Lehtinen and Walsh, 2011; Louvi and Grove, 2011; Johansson et al., 2013; Yeh et al., 2013). It is considered as a complex organelle that is retained by neuroepithelial cells and features of it are passed on to nearly all of its derivatives (i.e., cortical cells). Being a hot spot for transducing signals in CSF, the primary cilium is known to occupy a central position in the regulation of neural cell fate transition, migration, differentiation, and functional maturation of APs and their progenies (Guemez-Gamboa et al., 2014). As a result, primary cilium dysfunction has been widely implicated in the pathogenesis of certain brain disorders (Youn and Han, 2018).

Two complementary structures known to be associated with the primary cilium are the centrosome and ciliary membrane (Figure 2). At the base of the primary cilium is the structure called the basal body which is essentially made up of the older of the two centrioles formed at the S phase of the cell cycle (Figure 2; Louvi and Grove, 2011). During differentiative divisions this older or mother centrosome is preferentially inherited by daughter cells whose AP identity is to be maintained, while the newly synthesized centrosome is passed to the progeny destined to undergo differentiation (Figure 2). Given that the two centrosomes (old and new) form the poles of the mitotic spindle, it is conceivable that their disturbance may have a far-reaching effect on the proper establishment of cell polarity, microtubule organization, symmetry, and even fate transition during brain development (Huttner and Kosodo, 2005; Shitamukai and Matsuzaki, 2012; Camargo Ortega et al., 2019). Indeed, the ablation of factors known to control centrosome function can perturb the type and balance of neural progenitor differentiation (Wang et al., 2009; Paridaen et al., 2013).

The ciliary membrane is on the other hand closely linked to the older centrosome and together both are endocytosed during mitosis (Paridaen et al., 2013). Notably, during differentiative divisions the ciliary membrane is also asymmetrically inherited such that the daughter cell that keeps it also inherits the primary cilium and hence acquires the proliferative AP property of the parent cell, whereas the other daughter cell lacking the ciliary membrane becomes poised for differentiation (Paridaen et al., 2013). Clearly, the ciliary membrane stands to be an equally vital structural component of APs that participates in cell fate transition decisions, which precede or co-occur with delamination.

### Apical Junctional Complexes

Apical junctional connections are cardinal features of polarized epithelial cells, including APs (Figures 1, 2). They perform functions such as contributing to the formation of paracellular boundaries needed to compartmentalize tissue space (Van Itallie and Anderson, 2006), participating in the establishment and maintenance of apicobasal polarity, and also providing sites for signal transduction (Schneeberger and Lynch, 2004; Miyoshi and Takai, 2008; Marthiens et al., 2010). Principally, apical junctions are sites of mechanical support in conjunction with dynamic alterations in the actin cytoskeleton. AJs and tight junction (TJs) make up apical junctional complexes (Miyoshi and Takai, 2008). At the ultrastructural level, apical junctional complexes are made up of composite transmembrane proteins like cadherins, claudins, and nectins which appear as ordered structures that circumscribe the apical and lateral boundaries of cells at the apical domain (Miyoshi and Takai, 2008). As previously mentioned, many proteins that constitute the apical junction complex also perform roles in cell signaling. This is mainly because such apical junction proteins are able to make direct or indirect connection, via the actin cytoskeleton, with cytoplasmic proteins known to be downstream effectors in such signaling pathways (Hartsock and Nelson, 2008; Miyoshi and Takai, 2008). For example, there exists a cadherin-catenin-actin module in apical junctional complex functionality. Cadherins are known to promote cohesion of AP cells, especially aRGCs, therefore forming a major structural basis of the apical (luminal) surface architecture at the ventricle. Through their intracellular domain, cadherins are able to indirectly interact with the actin cytoskeleton via their connection with the α- and β-catenin in the cell cortex (Kobielak and Fuchs, 2004; Suzuki and Takeichi, 2008). It implies that cadherins and their cytosolic adaptor proteins may create a functional module that operates singularly or in concert with other factors to achieve the formation and maintenance of the apical junctional complexes to afford their mechanical and cell signaling functions.

During brain development, the modality and nature of inheritance of apical junctional complexes, especially AJs, are instructors of AP daughter cell fate acquisition and drive corticogenesis (Veeraval et al., 2020). In particular, the unequal distribution of such AJs among daughter cells of APs (Marthiens and ffrench-Constant, 2009) is a central predictor of their fate choice such that the progeny with less AJ association is unstable at the ventricular surface and acquires the tendency to differentiate and depart from the VZ as a basal cell (Figure 2). It implies that disturbance at or in the extracellular aspect of apical junction complexes formed by APs can have implications for abnormal signaling cascades in the intracellular milieu. Indeed, it has been reported that signaling pathways like Wnt and Rho are distorted following the ablation or overexpression of their related effectors like β-cat, RhoA, and Rac1 at the level of AJs, and lead to phenotypes that engender precocious cell delamination, aberrant cell proliferation, abnormal fate switch, and several neurodevelopmental anomalies (Chenn and Walsh, 2002, 2003; Junghans et al., 2005; Cappello et al., 2006, 2012; Costa et al., 2008; Katayama et al., 2011).

### Basolateral Membrane

The function and mode of inheritance of basal structures of APs also critically determine various cell biological properties of their progeny and are relevant in the regulation of cell delamination. One such key basal component of APs is the basolateral plasma membrane, which is sculpted as neuroepithelial cells transition to aRGCs (Figure 2). It makes up the bulk of aRGC plasma membrane and expands as cortical development advances. It can be divided into proximal and distal domains. The proximal segment, mainly found in the germinative zone of the developing cortex, has enough room to harbor the nucleus and permits its dynamic movement during interkinetic nuclear migration (Taverna and Huttner, 2010). Distally, the basolateral plasma membrane of aRGCs becomes slender and basally elongated, hence named the basal process. It traverses the rest of the cortical wall to make attachment with the basal lamina at the pial surface (Figure 1). Close to its basal anchorage, the basal process terminates as an endfoot with a club shape that can dynamically change to highly branched structures (Yokota et al., 2010).

The basal process and endfoot of aRGCs influence cell properties and processes like polarity, proliferation, migration, fate choice of daughter cells, and maintenance of overall tissue architecture [reviewed in Taverna et al. (2014) and Kon et al. (2017)]. A signaling niche exists in the vicinity of the basal lamina of APs that influences the phenotype of aRGCs during cortical development (Hartfuss et al., 2003; Radakovits et al., 2009; Seuntjens et al., 2009; Siegenthaler et al., 2009). Thus, the basal process may act as a conduit for the transmission of biochemical signals from the endfoot of the related aRGC soma or adjoining apical structures to orchestrate specific developmental events (Weissman et al., 2004; Tsunekawa et al., 2012; Pilaz et al., 2016; Rash et al., 2016).

During division of aRGCs, the basal process can either be symmetrically or asymmetrically inherited by daughter cells. With symmetric division, either the basal process is split into two and equally shared by daughter cells or one cell inherits all the parent basal process and the other cell grows its own basal process to make contact with the basal lamina, thus constituting proliferative division (Miyata et al., 2004; Kosodo et al., 2008). Perhaps the latter somehow manages to inherit the parental property of possessing a basal process. It would be interesting to elucidate the mechanistic bases thereof. Conversely, during asymmetric division, a daughter cell inherits all the mother basal process in order to maintain the pool and properties of the parent cell, whereas the other cell which inherits no basal process or the ability to develop one becomes differentiative and relocates basally as either BP or post-mitotic neuron (Miyata et al., 2004; Shitamukai et al., 2011).

## Mechanisms of Cell Delamination During Cortical Neurodevelopment

Several discrete events interact to trigger the process of cell detachment. As reviewed in Wilsch-Bräuninger et al. (2016), both extracellular and intracellular signals are integrated in the mechanisms that result in detachment of daughter cell(s) of AP from the VZ to acquire more differentiative fate (Wilsch-Bräuninger et al., 2016). Key among the mechanisms involved in delamination of anchored cells in cortical neurepithelium include, dismantling of the AJ belt, loss of cell polarity, basolateral ciliogenesis, post-mitotic repositioning of organelles, and migration of daughter cell(s). These salient mechanistic considerations are further discussed below.

### Remodeling of Apical Junctional Complexes

As previously discussed, APs in the cortical neurepithelium are typically integrated in the apical AJ belt (Veeraval et al., 2020). This means that basally fated derivatives of APs would have to withdraw from the anchoring AJ complexes to move to adluminal positions (Figure 2). It is possible that the protein components of AJ complexes may have to be remodeled to allow easy detachment of differentiating cells following cytokinesis of neuroepithelial cells (Figure 2). Thus, AJ proteins could be transformed into less tethering junctional complexes as observed during epithelial–mesenchymal transition (i.e., E-Cadherin to N-Cadherin) before the onset of cortical neurogenesis (Hatta and Takeichi, 1986; Dady et al., 2012) or modified into the so-called spot-like AJ complexes which favor BP biogenesis in the developing mammalian neocortex (Wilsch-Brauninger et al., 2012). It is also likely that such AJ proteins may be transcriptionally, epigenetically, or endocytotically downregulated to render the junctional belt sufficiently flimsy to aid cell detachment (e.g., Kawauchi et al., 2010; Itoh et al., 2013a; Das and Storey, 2014; Narayanan et al., 2015; Nguyen et al., 2018; Xie et al., 2019).

Interestingly, the mode of AP cell division influences how the AJ is distributed among daughter cells and appears to play key deterministic role in their delamination. Depending on orientation of the cleavage plane, the AJ complex may be divided into two or bypassed during AP cytokinesis (Delaunay et al., 2017). Proliferative AP cell division may come about as result a of equal distribution of AJ belt component, whereas certain asymmetric division can lead to one daughter cell collecting all the AJ belt complex to remain proliferative, while the other devoid of AJ belt goes into differentiation and delaminates (Figure 2; Matsuzaki and Shitamukai, 2015). Integrity of the AJ belt is crucial in regulating the movement and transduction of CSF-borne signaling factors capable of causing AP fate transition via modulation of the cell delamination tendency of the AP progeny (Chalasani and Brewster, 2011; Zhang et al., 2013; Hatakeyama et al., 2014; Shao et al., 2020). Similarly, it is conceivable that intracellular mechanisms may synchronously interact with paracellular extracellular cues to destabilize the AJ belt leading to loss of apical polarity and susceptibility to delamination.

### Loss of Apical and/or Basal Cell Polarity

As previously mentioned, the apicobasal polarity of APs underpins many of their cell biological behaviors. The interaction between AP cytoskeleton and adjoining AJ belt is critical for ensuring/maintaining the apical-basal polarity of progenitor cells in the cortical neurepithelium (Nagasaka et al., 2016; An et al., 2017), and a requisite for the delamination of their progenies (Kasioulis et al., 2017). The apical and basal domains established by the polarity of APs are unique signaling sites for regulating their fate maintenance and transition. However, many BPs and all neurons lack such domains, making them behave differently from their parent cells, the APs. It has emerged that prior to the differentiative cell division of APs to generate BPs, certain intracellular processes occur to influence the integrity of their apicobasal polarity and other subtle characteristics. Mechanistically, it is possible that the loss of apical polarity of APs may occur either through retraction from apical anchorage or basal attachment or both simultaneously. At least, it has been proposed that during apical anchorage disengagement of basal AP derivatives, the apical plasma membrane of newborn basal cells may be internalized or expelled as midbodies/ectosomes by endocytosis and exocytosis, respectively, to cause loss of apical polarity (Marzesco et al., 2005; Dubreuil et al., 2007). Moreover, the phenomenon of apical abscission – a notable mechanism that engenders cell delamination in neural tissue – which involves actin-myosin network contraction and pulsatile, also triggers loss of apical attachment in the VZ (Figure 2; Das and Storey, 2014; An et al., 2017; Simões et al., 2017). By extension, disruption of apicobasal polarity of APs can also emanate from molecular and structural alterations at their basal end or attachment. Together, loss of apical and/or basal polarity of APs has implication for the loss of the apical plasma membrane, primary cilium disorganization, cytoskeletal dynamics, and AJ belt remodeling which culminate in cell delamination in the VZ of developing cortex.

### Basolateral Ciliogenesis

Ciliogenesis, the establishment of a cilium, in vertebrate mainly involves formation of the basal body of the cilium via association of the older centriole with the plasma membrane, and development of the axonemes together with outgrowth of the ciliary membrane (Figure 2; Pedersen et al., 2008; Santos and Reiter, 2008; Rohatgi and Snell, 2010; Ishikawa and Marshall, 2011). According to Wilsch-Brauninger et al. (2012), the process of establishing a cilium on the basolateral aspects of the BP plasma membrane, i.e., basolateral ciliogenesis, is a pre-delamination event during AP fate transition. They specifically reported that during asymmetric mitotic division of AP cell, the mode of ciliogenesis exhibited by daughter cells is a determinant of which progeny stays as AP or switches fate to become BP (Wilsch-Brauninger et al., 2012). Typically, the primary cilium is endocytosed rather than disassembled, and wholly inherited together with its associated ciliary membrane by the daughter cell to become the AP progeny. This makes it possible to direct the primary cilium to the apical membrane domain to re-establish the apical primary cilium in the AP daughter cell. However, the presumptive BP takes up the newly formed centrosome which is without remnant of the ciliary membrane and drifts to the lateral plasma membrane to result in basolateral ciliogenesis (Wilsch-Brauninger et al., 2012). It is believed that the process of basolateral ciliogenesis comprises several mechanisms that contribute to the process of cell delamination. These include (i) promotion of the constriction of AJ belt, (ii) transduction of abventricular paracrine signals that may underline fate transition, and (iii) promotion of basal transport of centrosomes which supports basal mitotic activities (Wilsch-Brauninger et al., 2012; Paridaen et al., 2013; Taverna et al., 2016). Basolateral ciliogenesis thus encourages cell delamination, which in turn is prerequisite for basal cell generation known to be indispensable for cortical growth (Wilsch-Bräuninger et al., 2016; Kawaguchi, 2020).

### Migratory Kinetics

Differentiating cells that have successfully disengaged from the restrictive AJ belt gain capacity to actively move out of the VZ to settle in the SVZ if they are IPCs, or to reside mainly in the CP if neurons or glia (Figure 1). This migratory exit from the VZ can be considered as the final step involved in the process of delamination as APs differentiate to basal cortical cells. In general, delaminated cells are able to migrate out of the VZ by internally generated force afforded by actomyosin contractility or by the more predominate mode involving the motor action of microtubule dynamics (Itoh et al., 2013b; Kengaku, 2018; Minegishi and Inagaki, 2020). Also, external physical force garnered from motogens in the extracellular milieu contribute to the initial migration of some basal cell from the VZ (Itoh et al., 2013b). Intracellular kinetics accompany the overall migration of delaminated cells. For instance, the Golgi apparatus moves from apical to perinuclear and pericentrosomal positions (Taverna et al., 2016). Although not directly occurring in differentiating cells, interkinetic nuclear migration, which involves oscillatory movement of the nucleus in APs before a final return to the apical surface at M-phase to undergo mitosis (Figure 2), may contribute to the generation of the initial displacement force needed to kick start the migration of delaminating cells out of the VZ (Taverna and Huttner, 2010; Taverna et al., 2016). In the absence of all these factors needed to generate the necessary external and internal force to enable cell migration out of the neurepithelium, delaminated cells stagnate in the VZ or are mislocated. This makes them stay and differentiate at abnormal locations in the developing cortex; hence leading to aberrant cortical cytoarchitecture known to underlie many neurodevelopmental disorders discussed later in this review.

## Significance of Cell Delamination During Cortical Development

Given that the primary neural progenitors in the cortical epithelium give rise to the majority of neurons and glia in the neocortex, their ability to undergo delamination can be considered as a critical neurodevelopmental phenomenon in the developing cortex. In agreement, the importance of cell delamination during cortical morphogenesis is reflected by the several desirable consequences of the process, such as genesis of BPs, and promotion of neurogenesis, in the developing brain (Kawaguchi, 2020). In addition, there is evidence that cell delamination is central to the process of neuronal migration, and also strongly implicated in the generation of glia (astrocytes and oligodendrocytes) in the developing cortex. The next subsections give further account of the outcome or relevance of cell delamination in cortical development.

### Progenitor Pool Expansion and Maintenance

Maintenance of neural progenitor cells like aRGCs in the neurepithelium is largely determined by adhesive molecules and related factors that keep them anchored at the ventricular surface, and hence regulate their delamination (Rousso et al., 2012). The need for cortical tissue growth calls forth expansion of the progenitor pool therein, which is partly afforded by planar or oblique division of the neuroepithelial cells for AP self-renewal or delamination-mediated BP generation, respectively (Morin et al., 2007; Konno et al., 2008; Marthiens and ffrench-Constant, 2009; Shitamukai et al., 2011). It can also be argued that apical neural progenitor cells may regulate (inhibit) their own delamination or loss of anchoring proteins at the apical surface to prevent their premature differentiation, which can cause depletion of the progenitor cell niche in the cortical neurepithelium (Zhang et al., 2010; Rousso et al., 2012).

As previously mentioned, a prominent consequence of cell delamination which leads to amplification of the progenitor population in the developing cortex is the generation of neurogenic BPs from APs (Wilsch-Bräuninger et al., 2016). Cell delamination following downregulation of AJ-related molecules is a critical mechanism for BP generation from APs leading to the formation of the SVZ (Tavano et al., 2018). Given the cellular crowding in the VZ and hence space limitation for progenitor pool expansion therein, the AP–BP cell transition can be thought of as a developmental strategy to circumvent the restricted expansion of the progenitor pool within the VZ. In their abventricular or basal location, BPs have relatively more room to proliferative to amplify the progenitor pool needed to support cortical expansion during the active phase of brain development. This implies that if newly formed BPs are unable to delaminate properly and exit the VZ, they will accumulate in the apical aspect of the cortical wall. Such crowding of progenitors in the VZ can distort the developing cortical tissue architecture and homeostasis. Precocious or stalled delamination of differentiating cells in the VZ can lead to mislocation of BPs and their increased proliferation (Katayama et al., 2011; Nguyen et al., 2018).

### Promotion of Neurogenesis

Generation of neurons is a crucial normal outcome of cell delamination in the developing cortex which is stimulated by the cell differentiation program in the neurepithelium. Apart from directly leading to the generation of post-mitotic neurons, the process of cell delamination in the developing cortex invariable provides an excellent avenue to augment the neurogenic output of apical progenitors via generation of BPs as discussed in the previous section. This implies that cell delamination in the cortical neurepithelium effectively permits maximization of the number of neurons produced per unit time from an AP cell. It is believed that the delamination of daughter cells as post-mitotic neurons from APs constitute the direct mode of neurogenesis which is traditionally inadequate in furnishing all the neuronal cell population in the cortex, hence the reliance on the indirect (BP-dependent) mode of neurogenesis known to amplify the neuronal output (Götz and Huttner, 2005; Kriegstein et al., 2006; Cárdenas and Borrell, 2020).

When APs undergo asymmetric cell division, the daughter cell that inherits the larger spindle pole acquires neuronal fate and differentiates into a neuron (Paridaen et al., 2013; Delaunay et al., 2014). The process of cell delamination is believed to be by itself normally a trigger for neurogenesis and neuronal differentiation (Rousso et al., 2012; Arimura et al., 2020). As a result, preventing or overly promoting the process of cell delamination in the cortical neurepithelium can have implications for abnormal production of neurons during corticogenesis. For example, when APs aberrantly delaminate due to loss of AJ proteins, their hyperproliferation does not necessarily lead to neuronal hypernumeracy. Instead, the highly proliferating APs become more susceptible to apoptosis, and neuronal differentiation is reduced, leading to decreased neuronal output (Nguyen et al., 2018). In other accounts, however, loss of AJ proteins promoted cell cycle exit and differentiation of neural progenitor cells primed to delaminate (Cappello et al., 2006; Zhang et al., 2010). In certain contexts of AP delamination, detached aRGCs continue to proliferate as bRGC-like cells which normally have the capacity to increase neurogenesis (Penisson et al., 2019).

### Initiation and Progression of Cell Migration

During neocortical development, all basal derivatives of APs, especially nascent neurons, must first disconnect from their apical attachment before freely moving out of the VZ (Hatta and Takeichi, 1986; Noctor et al., 2004; Itoh et al., 2013a). Delamination thus appears to be the principal or ultimate mechanism to permit migration of differentiating cells in the neurepithelium. It is known that before the onset of migration of delaminated cortical basal cells, many fate commitment transcriptional mechanisms act in concert with factors that drive migration to equip cells with the capacity to exit their site of delamination to mainly settle in the (outer) SVZ as committed neurogenic precursor cells or in the CP as post-mitotic neurons (Pacary et al., 2012; Itoh et al., 2013b). On the other hand, the active force with which such delaminated cells migrate is provided by dynamics of the cytoskeleton. Cytoskeletal remodeling provides the required morphology and tensile force that permits active migration of delaminated cells. The physical link between AJs and the actin cytoskeleton underscores the important contribution of the process of cell delamination to cell migration, and the impact of dysregulating such connection in the developing cortex (Honda et al., 2003; Kobielak and Fuchs, 2004; Shen and Turner, 2005; Yamazaki et al., 2007; Suzuki and Takeichi, 2008; Nagasaka et al., 2016; An et al., 2017). It implies that during cell delamination, as the AJ complex is undergoing remodeling or disassembly, the actin cytoskeleton is stimulated to cause assembly of structural and regulatory proteins at the contact site that eventually leads to the needed cell morphology capacitation and the generation of the physical driving force required for the actual migration of the resultant delaminated cell (Miyoshi and Takai, 2008). The significance of unperturbed delamination for proper cell migration is partly supported by the observation that uncontrolled AP detachment, due to loss of AJ, may have contributed to failed/defective neuronal migration, cell clustering, and abnormal cell death in the VZ leading to aberrant cortical development (Itoh et al., 2013a; Nguyen et al., 2018; Sokpor et al., 2021).

### Regulation of Gliogenesis

If aRGCs or their progenies that have acquired gliogenic fate are unable to properly disengage from the AJ belt, production of glia in the developing cortex can be affected leading to an imbalance between the production of neurons and glia or abnormal aggregation and mislocation of same. For instance, if some of the previously discussed elements essential for cell delamination in the cortical neurepithelium (e.g., AJ belt formation, apicobasal polarity) are ablated, an increase in astrocyte production is observed, alongside their abnormal location in the cortex (Beattie et al., 2017; Jossin et al., 2017; Kiszka, 2019). By extension, because aRGCs also give rise to oligodendrocytes during mammalian neocortical development, it is logical that oligodendrogenesis may also be perturbed in the absence of normal cell delamination in the neurepithelium during the active phase of aRGC transformation or division to produce pre-oligodendrocyte precursor cells. Indeed, an account has been given where the abnormally developing cortex (due to BAF complex ablation) with loss of AJ complex and increased delamination of Pax6 or Sox2-expressing aRGCs (Nguyen et al., 2018), may have caused notable decrease in the number of OLIG2/PDGFRα-expressing oligodendrocyte precursor cells–and hence oligodendrocytes–in the early postnatal cortex of such mutants (Abbas et al., 2021). Moreover, it was observed that neural stem cells or aRGCs lacking anchorage and proper stemness in the VZ are forced to differentiate into oligodendrocytes at the expense of astrocytes (Niola et al., 2012). Clearly, a deeper interrogation of how cell delamination in the cortical neurepithelium affects the genesis of glia is needed to consolidate our understanding of the topic.

## Molecular Factors Regulating Neuroepithelial Cell Attachment And/Or Delamination

**FIGURE 3:**
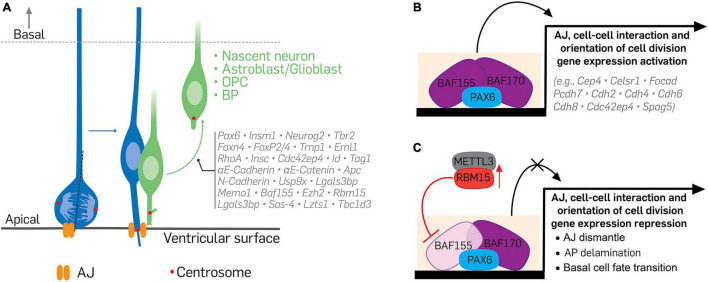
Illustration of the transcriptional and epigenetic regulation of delamination. **(A)** The delamination of differentiating cells from apical to basal aspect of the cortical wall is under the control of specific transcriptional and epigenetic programs during cortical development. Neural cells at the verge of differentiating activate or suppress certain molecular factors, which lead to their withdrawal from apical adhesion and subsequent exit from the germinal neurepithelium. **(B,C)** Indicate a typical epigenetic program capable of altering the transcriptome of apical progenitors or their progenies to affect how they delaminate during cortical development. Under normal condition **(B)**, recruitment of the BAF complex, via binding of its scaffolding subunits BAF155 and BAF170, to Pax6-bound regulatory elements promotes the expression of genes necessary for apical cell adhesion, proper orientation of mitotic spindle, and maintenance of the apicobasal polarity of cortical apical progenitors. Together, these preserve the proliferative nature of cortical neurepithelium. **(C)** However, to activate cell differentiation in the neurepithelium, BAF155 expression is downregulated or inhibited in neuroepithelial cells. A notable mechanism of BAF155 downregulation is inhibition of its expression by METTL3-mediated activity of RBM15 or by RBM15 upregulation. Lack of BAF155 alters PAX6-mediated BAF complex recruitment/engagement leading to downregulation of the genes involved in cell adhesion, cell division, and cell polarity maintenance. These culminate in cell differentiation, loss of adherens junctions, and increased delamination of apical progenitors or their derivatives. Vertical red arrow pointing upward denote upregulation or overexpressing of RBM15. AJ, adherens junction; AP, apical progenitor; BAF, Brg1/Brm associated factor; METTL3, methyltransferase like 3; RBM15, RNA binding motif protein 15.

Over the years, it has become clear that the process of delamination is a complex cell biological phenomenon involving many discrete regulatory circuits, which reflect the many factors that drive the event. Central among these factors is the transcriptional regulation of the AJs and cell-cell adhesion molecules that anchor APs in the VZ of the developing cortex (Figure 3A). A conceivable regulatory network involving epigenetic and chromatin-related factors, transcription activators and repressors, and other downstream molecular elements may interact to effect cell delamination as a major neurodevelopmental process during brain morphogenesis (Figure 3). In this section, we discuss notable factors involved in the apical attachment of cells and how they can regulate cell delamination in the cortical neurepithelium.

### Cell Adhesion, Division, and Polarity-Related Factors

Component molecules of AJs are vital for brain tissue morphogenesis (Harris and Tepass, 2010). Factors that ensure apical-basal polarity of cells, including those essential for cytoskeleton remodeling, in the cortical wall also co-function as cell adhesion molecules critical for cell attachment and, by extension, the regulation of delamination. Thus, the regulatory effects of cell adhesion and polarity-related factors can be considered as proximal to the initiation of delamination. Frequently, by downregulating the expression or remodeling dynamics of cell adhesion and polarity-related factors, APs or their basal derivatives are able to retract from the AJ-belt anchorage, leading to their basal relocation for cortical tissue histogenesis. As a result, improper control or lack of cell adhesion and polarity dynamics at the VZ can cause undesirable delamination and affect neurodevelopmental processes (Supplementary Table 1). The following examples indicate how such cell adhesion and polarity proteins are importance for the attachment of cells or their ability to detach in the cortical neurepithelium.

#### RHOA

Rho family GTPases have been identified as key regulators of epithelial apical junctional complexes formation and maintenance (Samarin and Nusrat, 2009). Such important roles highlight the Rho family GTPases as critical modulators of neuroepitheliogenesis. The small Rho GTPase RhoA, which localizes at AJs of cortical neurepithelium, can be considered as an essential regulator of cell delamination due to its key role in maintaining cell attachment. In addition, it is able to modulate effectors of hedgehog signaling and cell cycle progression during neural development (Katayama et al., 2011). AJs are disrupted when RhoA is lacking in the developing cortex (Katayama et al., 2011). In effect, Pax6-expressing AP delamination increases, and their cell cycle becomes defective, leading to abnormal expansion of the progenitor pool. These abnormalities grossly manifested as massive cortical dysplasia in the developing RhoA-mutant mouse (Katayama et al., 2011).

#### CDC42

CDC42 is another member of the Rho-subfamily which serves several functions in regulating signaling pathways and cell biological events, including cell cycle, migration, and morphology. The ventricular apical surface is one of the localization domains of CDC42-related proteins in the developing mouse neocortex, and likely gives reason to the observation that loss of CDC42 early in cortical development results in loss of AJs and attendant increase in aRGC delamination (Cappello et al., 2006; Georgiou et al., 2008). Promotion of BP genesis is an observable phenotype in the *Cdc42* mutant cortex (Cappello et al., 2006). Interestingly, the downstream CDC42 effector protein 4 (CDC42EP4) was also reported to elicit similar delamination phenotype following its shRNA-mediated silencing in the developing mouse cortical neurepithelium (Narayanan et al., 2018).

#### Cadherins and Catenins

It has been shown that certain factors known to contribute to formation of cadherin-catenin junctional complexes, which are concentrated at the apical surface of the neurepithelium (Gumbiner, 2005), are important regulators of cell delamination in the VZ (Veeraval et al., 2020). Those specifically identified to be crucial for delamination include N-Cadherin, αE-Catenin, E-Cadherin, β-Catenin, and αE-Cadherin, all of which are highly expressed at the cortical ventricular surface and can cause the AJ-belt to dismantle when downregulated (Supplementary Table 1). Loss of these cadherins and catenins in the developing cortical neurepithelium results in loss of apical polarity of aRGCs and resultant unnecessary delamination, leading to ectopic localization of neural progenitors, increase in neurogenesis, cortical laminar disorganization due to improper neuronal migration, and cortical hyperplasia (Junghans et al., 2005; Lien et al., 2006; Kadowaki et al., 2007; Stocker and Chenn, 2009; Das and Storey, 2014; Hatakeyama et al., 2014; Martinez-Martinez et al., 2016). Interestingly, in the absence of αE-Cadherin and αE-Catenin, the cell cycle of aRGCs is shorter, leading to increased cell cycle exit and cell survival (Lien et al., 2006; Stocker and Chenn, 2009). Conversely, β-Catenin ablation in the cortical neurepithelium resulted in increased apoptosis of the delaminated cells and likely without any obvious change in mitosis of the cortical progenitors therein (Junghans et al., 2005). It is possible that factors involved in the formation of the cadherin-catenin complexes may rely on cascading pathways such as β-Catenin, Notch, Hedgehog, and Reelin signaling in the regulation of delamination during cortical morphogenesis (Lien et al., 2006; Stocker and Chenn, 2009; Hatakeyama et al., 2014; Matsunaga et al., 2017).

#### LLGL1

As a major regulator of cell polarity, LLGL1, one of the two mammalian homologs of Lethal giant larvae (Lgl), was reported to connect the polarity of APs to cell-cell adhesion in the developing cortex (Klezovitch et al., 2004; Jossin et al., 2017). For this reason, deletion of *Llgl1* gene in embryonic neural stem cells resulted in severe loss of AJs leading to breakdown of cohesion among APs, loss of apicobasal polarity of APs, and disruption of the ventricular surface integrity (Jossin et al., 2017). The dislodged APs in *Llgl1* mutant brains are hyperproliferative and generate neurons that are unable to migrate properly, but instead accumulate and form ectopic neural mass which grows into the ventricular space to constitute periventricular heterotopia or subcortical band heterotopia (Beattie et al., 2017; Jossin et al., 2017). A cell-autonomous role of LGL1 in the production of neocortical astrocyte was also observed in *Lgl1* brain (Beattie et al., 2017). Analysis of how LGL1 interacts with cadherin-catenin proteins to maintain AP polarity and AJs at the ventricular surface revealed that it may achieve that via physically interaction with N-Cadherin, however, in an aPKC-mediated phosphorylation-dependent manner (Betschinger et al., 2005; Jossin et al., 2017). LGL1 is likely to act downstream of the Par3/Par6/aPKC complex to maintain cell polarity of APs (Betschinger et al., 2003). It is possible that to ensure delamination occurs temporally under controlled condition, LGL1 is centrally targeted for inactivation by phosphorylation (Betschinger et al., 2003) to regulate retraction of differentiating cells from the AJ-belt in the cortical neurepithelium. The aforementioned LGL1-Par3/Par6/aPKC complex axis may feature collateral regulatory inputs to control cell delamination. For example, CDC42, which regulates delamination (Cappello et al., 2006; Narayanan et al., 2018), may activate aPKC-mediated phosphorylation and inactivation of LGL1 at the apical membrane domain of APs (Betschinger et al., 2003, 2005; Plant et al., 2003) leading to AJ remodeling and delamination. Nonetheless, it should be interesting to find out the fine details of how aPKCλ silencing causes AJs to dismantle at the ventricular surface to cause delamination in the mouse neocortex (Imai et al., 2006). Perhaps, the physical association of aPKC with other AJ molecules at the ventricular surface (Manabe et al., 2002) is another means of ensuring correct AJ remodeling to permit physiological rate of cell delamination.

#### Cytoplasmic FMR1 Interacting Protein 1

The cytoplasmic FMR1 Interacting Protein 1 (CYFIP1) is enriched in the apical endfeet of aRGCs at the ventricular surface and involved in regulating AJs and apical polarity of aRGCs in the developing mouse cortex. Human neural progenitor cells lacking CYFIP1 display defects in AJs and apical polarity (Yoon et al., 2014). shRNA-mediated knockdown of *Cyfip1* in the mouse cortical neurepithelium resulted in ectopic localization of Pax6-expressing aRGCs due to loss of their apical attachment at the ventricular surface, and an increase in non-surface dividing PH3-expressing cells. In addition, IPCs and cortical neurons derived from CYFIP1-deficient aRGCs are ectopically located (Yoon et al., 2014). Together, these phenotypes pinpoint a critical role for CYFIP1 in maintaining cell attachment and by implication delamination. Mechanistically, given that CYFIP1 functions as a component of the WAVE complex in regulating cell polarity (Chen et al., 2010), it is plausible aRGCs depend on WAVE signaling, or at least its downstream effector Arp2/3, in controlling AJ dynamics at the ventricular surface to afford appropriate delamination of differentiating APs in the cortical neurepithelium (Yoon et al., 2014).

#### Inscuteable

The ability of APs to undergo symmetric division for self-amplification or divide asymmetrically to produce differentiated cells, including postmitotic neurons, can be determined by spindle orientation during mitosis of such polarized neuroepithelial cells (Yu et al., 2006; Buchman and Tsai, 2007). The adaptor protein inscuteable (INSC), which regulates spindle orientation during mitosis, is enriched at the apical surface of the developing mammalian cortex (Postiglione et al., 2011) due to the active division of APs/aRGCs in the VZ. By mediating the link between cell polarity and spindle positioning (Culurgioni et al., 2011; Yuzawa et al., 2011; Mauser and Prehoda, 2012), and possibly cell–cell adhesion coupling (Gloerich et al., 2017), INSC may act as a regulatory factor in the process of delamination. Indeed, it was observed that detachment and basal displacement of Pax6-expressing aRGCs in the VZ increases with INSC enrichment and promotes BP genesis and neuronal differentiation (Konno et al., 2008; Postiglione et al., 2011). The converse is true for INSC deficiency in the cortical neurepithelium, which leads to reduced neuronal differentiation partly due to reduction in oblique and vertical mitotic spindle orientation in dividing aRGCs (Postiglione et al., 2011) – explainable in the context of delamination downregulation.

#### LGN

Another factor which regulates spindle orientation in dividing APs and capable of influencing delamination is LGN (a.k.a. GPSM2), a G-protein regulator. It is highly expressed at the ventricular surface in the embryonic neocortex, and when deficient in cortical neuroepithelial cells spindle orientation becomes randomized, leading to induction of BPs at the expense of APs (Konno et al., 2008; Lacomme et al., 2016). Thus, increase in non-surface dividing progenitors (both Tbr2 and Pax6-expressing cells) in the mutant neocortex emanated from increased delamination of APs lacking LGN. It implies that LGN is necessary for proper division of aRGCs to ensure their self-renewability in the developing cortical neurepithelium (Morin et al., 2007; Konno et al., 2008). Given that INSC overexpression phenocopies LGN ablation in the embryonic mammalian cortex (Konno et al., 2008; Postiglione et al., 2011), and there is a competitive binding relation between them (Culurgioni et al., 2011), it would be interesting to interrogate the precise interplay between both factors in regulating spindle orientation during AP division in the germinal zone of the developing cortex.

### Transcription Activators and Repressors

While factors which form AJs or their association with the cytoskeleton at the apical surface have direct implication for cell delamination regulation, it has been demonstrated that transcriptional regulation is an essential and a first step in cell delamination. As such, some transcription factors including PAX6, SOX2, TBR2, INSM1, NEUROG2, and FOXP2/3, have been identified to control the attachment/delamination behavior of aRGCs or their progenies in the neurepithelium (Supplementary Table 1; Pacary et al., 2012). Such transcription regulators can generally be thought of as basically controlling the transcriptional program of cell delamination. Below, we discuss some of these transcription regulators or factors and their mechanistic determination of delamination.

#### Paired Box 6

The Paired box 6 (PAX6) transcription factor is a prominent regulator of many aspects of cortical development. It is highly expressed in APs and a subset of BPs, including bRGCs (Georgala et al., 2011; Ypsilanti and Rubenstein, 2016). Studies, including ours, have shown that the transcriptional regulation of delamination in the cortical neurepithelium is critically under the control of PAX6 transcriptional program (Figures 3A,B). Deletion or knockdown of Pax6 in neural stem cells in the developing cortex alters the transcription program of genes (e.g., *Cdc42ep4*, β*-catenin*, *Par complex*) whose proteins localize at the apical surface and largely participate in the formation of cortical AJ-belt (Asami et al., 2011; Narayanan et al., 2018). Loss of these gene targets due to Pax6 ablation thus led to AJ disappearance and increased delamination of APs in the developing neocortex (Figure 3C; Asami et al., 2011; Narayanan et al., 2018). APs that have lost Pax6 have increased tendency to divide asymmetrically to generate BPs probably as a result of the preponderance of non-vertical sub-apical cell division of the mutant APs (Asami et al., 2011; Narayanan et al., 2018). Altered expression of *Spag5*, a regulator of cell division orientation and direct target of Pax6, may underscore the promotion of BP genesis following Pax6 ablation in the cortical neural stem cells (Asami et al., 2011). Together, it is evident that delamination is under the control of Pax6 during cortical development.

#### Insulinoma-Associated 1

Insulinoma-associated 1 (INSM1) is a zinc-finger transcription factor which is expressed in the brain by mitotic neurogenic progenitors (APs and BPs). It is known for its role in BP biogenesis and results in BP pool depletion when inactivated in the developing mammalian neocortex. On the other hand, forced expression of INSM1 in APs induces their non-apical division and transition to BP (Farkas et al., 2008; Tavano et al., 2018). A remarkable mechanism by which INSM1 drives BP production is by regulating pre-delamination events such as basolateral ciliogenesis to make APs or their daughter cells acquire BP lineage and exit the neocortical neurepithelium (Wilsch-Brauninger et al., 2012). Further, it was observed that increased expression of INSM1 in APs caused them to aberrantly delaminate to become BPs, including bRGCs, due to a downstream repressive effect on Plekha7, a factor that associates with and stabilizes apical AJs in the developing mouse neocortex (Tavano et al., 2018). Upstream, NGN2 may be a regulator of INSM1 expression (Farkas et al., 2008; Tavano et al., 2018) to modulate the differentiation of APs to BPs via control of delamination. In this case, it is possible that INSM1 expression is suppressed by NGN2 downregulation to maintain the cortical neurepithelium or cause the APs therein to differentiate and delaminate under the influence of INSM1 enrichment following NGN2 upregulation. Of note, TBR2 and RND2, both induced by NGN2 may cooperate with INSM1 to effect delamination events, where TBR2 plausibly co-functions as a pre-delamination effector (Arnold et al., 2008; Sessa et al., 2008) and RND2 causes the exit of delaminated cells from the VZ via cell migration (Heng et al., 2008).

#### SCRATCH1/2

The expression of the zinc-finger transcription factors SCRATCH1 and SCRATCH2 (SCRATCH1/2) is prominent in neurons and BPs, causing their enrichment in the SVZ, intermediate zone, and cortical plate of the developing and adult cortex (Marín and Nieto, 2006; Itoh et al., 2013a). SCRATCH1/2 expression coincides with neuronal fate commitment under the control of proneural factors like NEUROG1 and ASCL1, and they regulate the onset of radial neuronal migration in the developing cortex (Itoh et al., 2013a). This brings into focus their involvement in the process of cell delamination which precedes the initiation of neuronal migration. Indeed, it was observed that expression (upregulation) of SCRATCH1/2 is required for cell detachment at the apical surface of the VZ before driving the migration of such delaminated cells. However, in the absence of SCRATCH1/2 progenies of APs committed to basal cell fate retained their apical attachment and were unable to exit the VZ, indicating failed delamination (Itoh et al., 2013a). Mechanistically, SCRATCH1 may cause remodeling of AJs by repressing the expression or reducing the apical enrichment of E-cadherin in the developing mouse neocortex leading to induction of delamination, initiation of migration, formation of the SVZ, and expansion of the cortical plate (Itoh et al., 2013a; Hatakeyama et al., 2014; Martinez-Martinez et al., 2016).

### Epigenetic and Chromatin Remodeling Factors

Various epigenetic and chromatin regulators are known to control neurodevelopment and neural function. Emerging evidence indicate how these factors regulate cortical morphogenesis through acting as indispensable determinants of delamination, which affects neurogenesis and gliogenesis in the developing cortex. These epigenetic factors can have direct effect on the transcriptional program, or the synthesis of protein factors known to modulate cell delamination. In this section, we describe the role of such epigenetic factors that operate at a higher level of gene regulation to control cell delamination during cortical formation.

#### BAF155

BAF155 is one of the scaffolding subunits of the chromatin remodeling BAF complex, which is the mammalian version of the SWI/SNF. While BAF155 is ubiquitously expressed, its expression is upregulated in certain cell types in a spatiotemporal manner. In the developing cortex, its expression in aRGCs peaks at mid-gestation, and largely colocalizes with PAX6, making the neocortical VZ strongly delineated by BAF155 immunostaining (Narayanan et al., 2018). It was revealed that reduction or lack of BAF155 expression in cortical APs may be a molecular requirement for aRGCs to undergo delamination and produce bRGCs. This is backed by the observation that deletion of BAF155 in the developing mouse neocortex led to loss of AJs at the ventricular surface that resulted in heightened aRGC delamination to produce BPs. Interestingly, such BAF155 mutant mouse cortex displays changes in the cortical transcriptome similar to the cortex lacking PAX6, and shows a potentiating effect on the transcriptional activity of PAX6 (Narayanan et al., 2018). Thus, it is possible that, at least, BAF155 cooperates with PAX6 to regulate the expression of genes involved in AJ formation and cell-cell adhesion (Figure 3B), and gives reason to the overlap between the delamination and bRGC-like cell genesis in the BAF155 knockout cortex compared with the PAX6 knockout cortex (Narayanan et al., 2018). Because sustained PAX6 expression alone can also cause increased cortical BP genesis likely associated with high frequency AP delamination (Wong et al., 2015), it would be interesting to find out how PAX6 overexpression impacts BAF155 expression in the scheme of delamination regulation. Perhaps, other layers of molecular regulation may be involved in the BAF155–PAX6-mediated BP genesis, especially in the context of bRGCs generation in the gyrencephalic cortex. A plausible collateral mechanism is the involvement of components of the polycomb repressive complex 2 (PRC2) which has a reciprocal antagonistic relationship with the BAF complex in (neural) tissue development (Boyer et al., 2005; Kadoch et al., 2017).

#### BAF170

BAF170 is the other scaffolding subunit which cooperates with BAF155 in organizing and stabilizing the BAF complex. It is apparent that the induction of BAF170 expression in embryonic stem cells for their commitment to neural progenitor cell lineage (Lessard et al., 2007; Ho et al., 2009) is essential for the regulation of indirect neurogenesis (Tuoc et al., 2013) partly through control of delamination in the neurepithelium during cortical development (Narayanan et al., 2018). High dose of BAF170 at the expense of BAF155 favors the delamination of aRGCs or their transition to bRGC-like cells by means of delamination (Figure 3C). This observation was made in the developing mouse cortex following BAF170 over expression (Narayanan et al., 2018), which is known to competitively reduce the amount of BAF155 in the BAF complex to drive or modulate BP genesis (Tuoc et al., 2013). Mechanistically, BAF170 may regulate BP generation via recruitment of REST-corepressor complex to the promoters of Pax6 downstream targets, including AJ genes, involved in cortical development (Tuoc et al., 2013).

#### Enhancer of Zeste Homolog 2

Interestingly, the catalytic subunit of the PRC2 called enhancer of zeste homolog 2 (EZH2), regulates installation of the gene silencing mark H3K27me3 (trimethylated histone H3 at lysine 27) in cortical neuroepithelial cells to ensure proper generation of their progenies (Pereira et al., 2010; Piper et al., 2014). Deletion of EZH2 in the developing cortex abolishes EZH2-mediated gene suppression leading to an abnormal increase in gene expression that cause distortion of the molecular program required to maintain a balance between AP proliferation and basal cortical cell (BPs and neurons) generation. As a result, the neurogenic phase of corticogenesis is limited in EZH2-null cortex, although EZH2 deficiency in APs seem to cause overproduction of BPs and neurons at the expense of aRGCs proliferation during early cortical development (Pereira et al., 2010). Even though the ventricular surface of the EZH2-null cortex appears unperturbed, it is possible that an increase in AJ remodeling that favors BP genesis may have caused an increased in early neurons and BPs, and the premature exhaustion of the PAX6-expressing AP pool. It is also logical that the early onset or acceleration of gliogenesis in the EZH2-null cortex led to the loss of the such neurogenic APs (Pereira et al., 2010), which can be explained by aberrant acquisition of glial fate by the mutant aRGCs and/or the rampant delamination of their gliogenic cell derivatives in the developing cortex. An investigation of AJ changes and delamination in the EZH2-null cortical neurepithelium is needed to consolidate this line of reasoning. Such delamination investigation would help reconcile the early onset of gliogenesis and limited neurogenic phase phenotype in the absence of EZH2 (Pereira et al., 2010), with the observation that EZH2 or PRC2 is essential for promotion of astrogenesis and suppression of neurogenesis (Hirabayashi et al., 2009).

#### RNA Binding Motif Protein 15

The RNA binding motif protein 15 (RBM15), which is a key component of the m^6^A methylation machinery, was identified as an important factor for cortical development through regulating cell delamination in the neurepithelium (Xie et al., 2019). Whereas cortical cells generally express RBM15, its level is dynamic in aRGCs; the significance of which is such that at higher levels it promotes the detachment of differentiating cells in the cortical neurepithelium, including PAX6-expressing bRGC-like cells, through loss of AJ (Xie et al., 2019). Interestingly the upregulation of RBM15 in a subset aRGCs coincides with a decreased amount of BAF155 levels in such population of aRGCs. Observation at the transcriptome level showed that RBM15 causes BAF155 downregulation via targeting it mRNA for METTL3-mediated methylation and subsequent degradation. The converse is true where downregulation of RBM15 in the neurepithelium results in upregulation of BAF155 (Xie et al., 2019). It implies that the BAF155-dependent transcriptional program which leads to the expression of protein factors essential for the regulation of cell delamination is distorted in the cortical neurepithelium when RBM15 is upregulated (Figure 3C). We think that an RBM15-BAF155 regulatory axis may be featured as part of the mechanisms which drive proper cell delamination to afford normal cortical neurodevelopment.

### Other Molecular Modulators of Delamination During Corticogenesis

Several other factors such as TRNP1, PDGFRβ, MARCKS, TAG-1, Lamin-B, ID, LZTS1, PFN1, TBC1D3, and APC that are expressed at the ventricular surface, around or in APs, have also been reported to be essential for the regulation of cell delamination during corticogenesis (Supplementary Table 1; Lyden et al., 1999; Weimer et al., 2009; Yokota et al., 2009; Kim et al., 2011; Okamoto et al., 2013; Stahl et al., 2013; Lui et al., 2014; Ju et al., 2016; Kawaue et al., 2019; Kullmann et al., 2020; Kerimoglu et al., 2021; Penisson et al., 2021). When abnormally expressed, these additional factors which are usually downstream transcriptional or epigenetic effectors of delamination have been identified to cause defective delamination-related phenotypes such as detachment and dispersion of APs from the VZ, aberrant fate transition of delaminated progenies of APs, and abnormal migration of newborn neurons. Of note, while mutation of some of such factors cause drastic AJ loss to trigger delamination, others lead to subtle AJ changes when ablated. A typical example is the factor EML1, which when misexpressed causes severe abnormal cortical phenotype due to increased cell delamination, which may not be explained by drastic AJ loss (Kielar et al., 2014; Uzquiano et al., 2019; Markus et al., 2021). Dysregulation of the delamination regulator USP9X is also known to result in abnormal delamination outcomes due to transient disruption of cell adhesion (Premarathne et al., 2017).

In the developing cortex, it was observed that AKNA regulates neurogenesis through keeping in check the delamination of newly formed BPs in the VZ via organization of centrosomal microtubule in neural stem cells, dissolution of anchoring cell adhesion and junctional complexes, and the constriction of apical endfoot of the RGC progeny with BP fate (Camargo Ortega et al., 2019). It is possible that CAMSAP3 recruitment by AKNA to centrosomes is a proximal axis for effecting delamination through orchestrating destabilization of microtubules at the AJ belt, leading to apical endfeet constriction at the ventricular surface (Meng et al., 2008; Tanaka et al., 2012; Pongrakhananon et al., 2018; Camargo Ortega et al., 2019). Lack of AKNA, however, leads to retention of AJs and prevents delamination of AP, leading to accumulation of basal derivatives that should normally move out of the VZ (Camargo Ortega et al., 2019). Interestingly, the migration of cells from the SVZ to the cortical plate is also under the control of AKNA by means of multipolar-to-bipolar transition regulation. As such, multipolar cells in the SVZ that lack AKNA are able to transition faster to bipolar morphology. However, such transition is hampered when AKNA is overexpressed in the multipolar cells, making them migrate improperly and accumulate in the SVZ (Camargo Ortega et al., 2019).

We are of the opinion that these observations imply the extent AJ loss or changes needed to cause detachment of cells is contingent on other regulatory inputs to achieve the needed mode and degree of delamination. This brings into debate whether there are various types of delamination in the developing cortex. Could it be that the delamination that occurs early in cortical development is different from that occurring at mid- or late-corticogenesis? Further studies focused of this subject are required to address it. For now, we can speculate that the mechanism afforded by several of the identified additional downstream effectors of delamination (Supplementary Table 1), including controlling pertinent processes like cell polarity and division (e.g., TAG-1, LZTS1, SAS-4, and APC), spindle orientation (e.g., Lamin-B), migration (e.g., MARCKS, Lamin-B, and LZTS1), microtubule assembly and stability (e.g., MEMO1 and PFN1), and cell adhesion (e.g., LGALS3BP and ID), can be coupled with the mechanism of AJ remodeling and primary cilium signaling to describe cell delamination on a spatiotemporal basis. Our understanding of delamination can also be expanded through the identification of the upstream transcriptional and epigenetic programs which determine the expression and activity of proximal mediators of delamination.

## Implicating Effects of Defective Cell Attachment or Dysregulated Delamination in the Pathogenesis of Brain Disorders

The delicate nature of cell delamination and the multiplex of regulatory factors involved give an indication of how detrimental improper detachment of cells in the VZ can be to the developing brain. Our literature search has revealed that abnormal (increased or decreased) cell delamination seems to be a common underlying pathophysiological process in the etiology of certain neurodevelopmental and neurological disorders characterized by a broad spectrum of cortical structure and function anomalies (Table 1). In general, when cells delaminate abnormally in the VZ the consequence is one of the following defective conditions or a combination of them which lead to specific or syndromic cortical disorders: (i) depletion of progenitor pool and reduction in neurogenesis, (ii) defective localization of progenitors and migration of differentiating cells, (iii) excessive increase in progenitor pool, (vi) lack or overproduction of glia, and (v) compromised ventricular surface integrity. In this last section of the review, we briefly discuss examples of brain (cortical) disorders which hinge on the said outcome(s) of unregulated delamination as notable pathogenic mechanism(s).

**TABLE 1:** Neurological and neurodevelopmental disorders associated with dysfunction of delamination-related factors.

Brain disorder	Experimental system	Factor(s) implicated	References
Heterotopia	Human, Mouse	EML1, LLGL1, RHOA, PARD3, LIS1	Sicca et al., 2003; Kielar et al., 2014; Jossin et al., 2017; Liu et al., 2018; Uzquiano et al., 2019; Markus et al., 2021
Intellectual disability	Human, Mouse	USP9X	Homan et al., 2014; Reijnders et al., 2016; Premarathne et al., 2017
Exencephaly	Mouse	MARCKS	Stumpo et al., 1995
Autism	Human	MEMO1	Nakagawa et al., 2019
Neurodevelopmental delay with intellectual disability	Human, Mouse	BAF170	Tuoc et al., 2013; Tuoc et al., 2017; Machol et al., 2019
Macrocephaly (megalencephaly)	Mouse	β-catenin, BAF170, PARD3	Chenn and Walsh, 2002; Tuoc et al., 2013; Liu et al., 2018
Pachygyria	Human	CTNNA2	Schaffer et al., 2018
Microcephaly	Human, Mouse	TBR2, WDR62, ASPM, SAS-4	Bond et al., 2005; Baala et al., 2007; Arnold et al., 2008; Insolera et al., 2014; Jayaraman et al., 2016
Schizophrenia	Human	CYFIP1	Yoon et al., 2014; Nebel et al., 2016
Hemorrhagic hydrocephalus	Mouse	LGL1	Klezovitch et al., 2004
Complex brain malformations	Human, Mouse	α-Catenin, PAX6, LGALS3BP	Bamiou et al., 2004; Lien et al., 2006; Schaffer et al., 2018; Kyrousi et al., 2021
Chudley-McCullough syndrome	Human	GPSM2 (LGN)	Doherty et al., 2012
Lissencephaly	Human	PAFAH1B1 (LIS1)	Lo Nigro et al., 1997; Pilz et al., 1998; Fogli et al., 1999

### Depletion of Progenitor Pool and Reduction in Neurogenesis: Microcephaly

When APs detach from the VZ in an unregulated manner, it can lead to depletion of the BP reserve, which eventually translates into decrease in cortical neurogenesis. Reduced cortical neurogenesis or loss of neuroglia due to apoptosis upregulation are central causes of cortical mass/tissue loss leading to small brain size, which phenotypically defines the condition called microcephaly. The microcephalic brain classically presents with functional deficits because of loss of neurons and neural connections. The delamination-promoting factor TBR2 has been implicated in the development of microcephaly (Supplementary Table 1; Baala et al., 2007; Arnold et al., 2008). Lack of TBR2 expression in differentiating APs prevents their delamination-driven conversion into BPs resulting in perturbation of SVZ formation and overall disturbance of cortical neurogenesis (Sessa et al., 2008), which can culminate in malformative microcephalic syndromes in human (Baala et al., 2007). The identification of WDR62 and ASPM as functional interaction partners in regulating AJs to affect delamination and as drivers of cortical neurogenesis, make them key determinants of brain size, and hence causative of microcephaly when ablated in the developing mouse and human cortex (Bond et al., 2002; Jayaraman et al., 2016). It is possible to expand the molecular causal agents involved in microcephaly pathogenesis on the basis of their involvement in cell delamination given the many more delamination regulator that engender loss of cortical parenchyma when misexpressed (Supplementary Table 1).

### Defective Localization and Migration of Cortical Cells: Heterotopia, Complex Brain Malformations, Neuropsychiatric Disorders

When anchored APs or their differentiating daughter cells delaminate abnormally, they commonly mislocate in the cortical wall partly due to aberrant migration. Such misplaced APs or their neurogenic progenies proliferate and/or differentiate ectopically to produce cortical cell or tissue mass that are abnormally located and leads to defective formation of neural connections and resultant behavioral deficits in the brain. Heterotopias (e.g., subcortical band heterotopia, periventricular heterotopia) and complex brain malformations are notable brain disorders in the aforementioned context of delamination dysregulation (Bamiou et al., 2004; Kielar et al., 2014; Reijnders et al., 2016; Jossin et al., 2017; Schaffer et al., 2018). Genetic analyses in mouse and human revealed that the cell attachment/delamination regulators RHOA, PARD3, EML1, and LLGL1 are involved in the pathogenesis of heterotopias (Table 1). The mislocation, disorientation, and change in mode of division of abnormally delaminated mammalian APs due to lack of EML1 and LLGL1 have been largely identified to cause the ectopic cortical tissue formation characteristic of heterotopias (Kielar et al., 2014; Jossin et al., 2017). The transcription factor PAX6 and AJ-related molecule α-Catenin are also implicated in the development of complex cortical malformations, likely triggered by aberrant cell delamination in mutant brain (Bamiou et al., 2004; Lien et al., 2006; Schaffer et al., 2018). Extracellular matrix factors which can regulate the delamination of neural progenitors can also cause complex cortical malformations when ablated. A case has been made for the involvement of the extracellular factor LGALS3BP, which normally regulates the anchorage and position of cortical progenitors, in the etiology of complex cortical malformation due to its *de novo* mutation (Kyrousi et al., 2021).

While it is expected that the heterotopic and severely malformed cortex may display behavior deficiency phenotypes, it is also possible that certain neurological conditions can stem from excessive cell delamination and attendant abnormal cell placement in the cortex without striking brain abnormality. Conditions like schizophrenia and some forms of intellectual disability are among the categories of brain disorders that may be caused by subtle consequences of abnormal cell delamination owning to improper control of neural progenitor cell or differentiating cell detachment in the developing cortex. This assertion is, in part, based on a genetic risk modeling experiment for schizophrenia in which dysfunction of the AP adhesion and polarity factor CYFIP1 led to delamination and ectopic localization of aRGCs (Yoon et al., 2014; Nebel et al., 2016). In the absence of CYFIP1, its epistatic interaction with ACTR2 (a mediator of WAVE signaling) is abolished, leading to susceptibility of neuropsychiatric disorders, including schizophrenia (Yoon et al., 2014). Another cell delamination regulating factor whose normal function may guard against neuropsychiatric disorders is USP9X. This is because certain individuals with USP9X mutations show intellectual disability and neurobehavioral deviations partly linked to abnormal neuronal migration and placement (Homan et al., 2014). It is possible that defective ciliogenesis in APs due to primary cilium signaling disruption in the absence of USP9X localization in primary cilium (Reijnders et al., 2016) may be a major pathogenic mechanism involved in the development of neuropsychiatric disorders caused by USP9X mutations. The observation that loss of USP9X in the developing mouse cortex disturbed AJs and polarity of APs leading to precocious BP genesis and their ectopic localization (Premarathne et al., 2017), provides further basis for the role of dysfunctional USP9X in the pathogenesis of certain neuropsychiatric disorders.

### Excessive Increase in Progenitor Pool: Megalencephaly

The non-physiological expansion of the progenitor pool in the developing cortex due to abnormal neural progenitor delamination and concomitant hyperproliferation of APs and/or their BP derivatives can have implication for increased production of the neuroglia, particularly neurons, that can result in enlarged cortical size or megalencephaly. The enlarged brain invariably causes abnormal head size (Macrocephaly) development and can result in neurodevelopmental and neurological deficits. Knockout of BAF170 in the developing mouse cortex was observed to cause excessive growth of the brain due to overproduction of BPs (Tuoc et al., 2013), which was likely engendered by altered cell delamination. In human, the brain with BAF170 mutation presents with neurodevelopmental and neurocognitive deficits (Machol et al., 2019), although an association with macrocephaly/megalencephaly is yet to be reported in such individuals. It is possible that the competitive interaction between BAF170 and BAF155 may elicit a ventricular surface alteration leading to AJ dynamics to increase delamination of AP progenies in the VZ. An obvious enlargement of the cerebral cortex due to increase in progenitor pool was also observed when the putative delamination factor PARD3 was ablated in the developing mouse cortical neurepithelium (Liu et al., 2018), therefore making PARD3 misexpression a potential macrocephaly causative factor. Another cell attachment regulator which is directly involved in AJ formation and can cause abnormal expansion of the cerebral cortex is β-catenin. When β-catenin is overly activated in the developing cortex neural progenitor cells become hyperproliferative resulting in increased progenitor cell population and differentiation of neuronal precursors (Chenn and Walsh, 2002).

### Overproduction or Lack of Glia: Brain Tumors, Neurodegenerative Disorders

Since aRGCs are a major source of cortical glia, their abnormal delamination can affect gliogenesis. Indeed, in the absence of factors like BAF155 and BAF170, a striking consequence is the overproduction of astrocytic progenitors and astrocytes in the developing mouse cortex (Kiszka, 2019). Increase in astrogenesis was also reported in the RHOA-ablated developing cortex (Katayama et al., 2011). Hence it is conceivable that astrocyte tumorigenesis (gliomas) can emanate from the dysregulation of the aforementioned delamination-associated factors in the developing forebrain. It is also possible that scanty number of generated astrocytes can result from aberrant expression of cell attachment/delamination factors, for example Id (Niola et al., 2012), which can impair neural function. In the same line of reasoning, the production of oligodendrocytes in the developing cortex can be affected when certain factors involved in delamination are ablated. It is expected that the disturbance of the oligodendrogenesis program in the developing mouse cortex due to loss of BAF155 and BAF170 (Abbas et al., 2021), lack (Niola et al., 2012) or overproduction (Kiszka, 2019) of cortical astrocytes will increase susceptibility to neuronal degeneration known to call forth neurodegenerative disorders. This proposed pathologic outcome implicating abnormal delamination of aRGCs remains to be explored.

### Compromised Ventricular Surface Integrity: Hydrocephalus

The AJ belt makes critical contribution to the stability and integrity of the junctional complex holding the monolayer ependymal cells at the cerebral ventricular surface (Figure 1; Sarnat, 1995). Therefore, unregulated loss or depletion of AJs due to abnormal cell delamination in the VZ can have implication for compromised cortical ventricular surface integrity emanating from defective transition of aRGCs to ependymal cells or delamination of the latter. Improper formation of the ependymal cell layer or injury to ependymal cells can result in some anomalies, including ependymal cell atrophy, interruption of the ependymal epithelium continuity, subventricular gliosis, inflammation, and hemorrhage (Sarnat, 1995; McAllister et al., 2017). Hydrocephalus is a common brain anomaly caused by defective ependymogenesis (Banizs et al., 2005; Domínguez-Pinos et al., 2005). Because improper delamination of neuroepithelial cells can disturb the pool of aRGCs which produce or later transition into ependymal cells, it is possible that factors which regulate AP anchorage and detachment in the developing cortical neurepithelium may have a role to play in the pathogenesis of hydrocephalus. A typical example is the cell polarity regulator LGL1. In the developing LGL1-null mouse cortex, the neuroepithelial cell population abnormally expands, and are susceptible to apoptosis. There is overt loss of AP cell polarity and AJs at the ventricular surface of the LGL1 mutant cortex (Klezovitch et al., 2004). These observations point to defective cell detachment in the mutant VZ and likely underlie the dramatic hydrocephalus phenotype displayed by the LGL1 mutants (Klezovitch et al., 2004).

## Concluding Remarks

During embryonic development of the cerebral cortex, it is essential for APs poised to differentiate to withdraw from the proliferative neurepithelium to ensure tissue homeostasis and appropriate cortical histogenesis. Thus, cell delamination has been identified as a neurodevelopmental mechanism crucial for the maintenance of the balance between the rate of neural progenitor cell proliferation and differentiation, and the coordination of cell fate with cell positioning or polarity. In essence, cell delamination is vital for proper neurogenesis, gliogenesis, neuronal migration, neural cell-type diversity, and neural plasticity. Therefore, the phenomenon of cell delamination has been proposed to be key in the establishment of proper brain structure and function. Aberrant neural progenitor or differentiating cell delamination–whether in excess, reduced, or untimely–can engender disturbance of neuroepithelial cell stemness, alter mitotic activity, and perturb cell polarity. These abnormal changes can precipitate precocious differentiation of cortical progenitors into neurons or glia in a disproportionate manner or preclude neuroprogenitor differentiation, leading to cortical malformations. Indeed, some brain disorders, including heterotopias, microcephaly, hydrocephalus, schizophrenia, and Chudley-McCullough syndrome have been reported to have misregulation of cell attachment/delamination-related factors as notable contributive elements in their pathogenesis. The multiple neurodevelopmental abnormalities which occur when neuroepithelial cell delamination goes awry highlight the need for precise and multi-level regulation of the process during cortical development. Indeed, we have come across a host of molecular factors involved in the regulatory circuit for ensuring proper cell delamination. Majority of the regulatory factors are critical for modulating discrete processes entailed in cell attachment and delamination namely cell adhesion, spindle orientation, cell division, primary cilium signaling, and cell polarity. It is apparent that certain transcription and epigenetic factors have indispensable roles in the regulation of delamination. We posit that the many factors identified as regulators of cell attachment/delamination can be considered as potentially causing novel or uncharacterized brain disorders. To expand the list of factors involved in cell attachment/delamination, we think future studies should aim at exploring factors known to cause delamination of differentiating cells in other tissue epithelia for possible replicative testing in the cortical neurepithelium. Also, consideration should be given to investigating factors involved in tumor formation and/or malignancy for their physiological role in delamination during cortical morphogenesis.

An interesting perspective is whether there can be a way to remedy excess delamination to stave off or minimize its impact on cortical structure and function. To that end, inspiration can be drawn from the observation that lateral adhesion can afford reintegration of cells which have improperly withdrawn from epithelial monolayers (Bergstralh et al., 2015). This reasoning or proposal indeed provokes a debate on whether there is the possibility of rescuing abnormally delaminated cortical neuroepithelial cells via treatment with exogenous factors that would promote the reintegration of aberrantly detached apical cells. We believe the stimulus to attempt such investigation is in the promise that its outcome would enrich current therapeutic opportunities targeting treatment of pertinent cortical disorders.

## Author Contributions

All authors listed have made a substantial, direct, and intellectual contribution to the work, and approved it for publication.

## Conflict of Interest

The authors declare that the research was conducted in the absence of any commercial or financial relationships that could be construed as a potential conflict of interest.

## Publisher’s Note

All claims expressed in this article are solely those of the authors and do not necessarily represent those of their affiliated organizations, or those of the publisher, the editors and the reviewers. Any product that may be evaluated in this article, or claim that may be made by its manufacturer, is not guaranteed or endorsed by the publisher.
