# Supplementary material for: Regulation of Cell Delamination During Cortical Neurodevelopment and Implication for Brain Disorders
Source: Front Neurosci. 2022 Feb 23;16:824802. doi: 10.3389/fnins.2022.824802 (PMC8904418; doi:10.3389/fnins.2022.824802)
Supplement: Supplementary file 1 [file Table_1.pdf]

Table 1. Molecular determinants of cell delamination in the developing cortical neuroepithelium				
**Factor**	**Mutant**	**Phenotype**	**Cofactor/ Effector/Target**	**Reference**
**Cell adhesion and polarity-related factors**				
RHOA	Foxg1-Cre, $RhoA^{fl/fl}$	AJ disruption, cortical dysplasia, abnormal expansion of progenitor pool, upregulation of hedgehog signaling, exencephaly-like protrusions	GLI1, FGF15, HES5, HES1, CyclinD1	(Katayama et al. 2011)
INSC	$Insc^{fl/fl}$, Nes-Cre	Deceased oblique and vertical mitotic spindles in dividing aRGCs, reduced neuron differentiation	Not determined	(Postiglione et al. 2011)
	$Insc^{ki/ki}$, Nes-Cre	Increased oblique and vertical mitotic spindles in dividing aRGCs, promoted aRGC delamination, increased generation of BP, increased neurogenesis and cortical thickness		
CDC42	$Cdc42^{fl/fl}$, Emx1-Cre, hGFAP-Cre	Loss of AJs, defective apically directed interkinetic nuclear migration, encouraged AP delamination and aberrant location of cortical progenitors, caused increase in BP fate acquisition by dividing APs	ARP2/3	(Cappello et al. 2006; Georgiou et al. 2008)

β-Catenin	β-catenin$^{fl/fl}$, FoxG1-cre	Loss of AJ-belt, caused abnormal delamination leading to breakdown of neuroepithelial structure, delaminated cells showed increased apoptosis, resulted incortical hypoplasia	Not determined	(Junghans et al. 2005)
CDC42EP4	Cdc42ep4 KD with shRNA	Promotes delamination of Pax6-expressing APs, Increases BP genesis	PAX6	(Narayanan et al. 2018)
LLGL1 (Lgl1)	Lgl1$^{-/-}$, Lgl1$^{fl/fl}$, Nestin-Cre, Emx1-Cre, Emx1-CreER	Loss of AJ and apicobasal polarity of aRGCs, defective aRGCs delamination and cell migration, activation of aRGCs proliferation, increased apoptosis of neural progenitors, increased astrogenesis, notch signaling upregulation, periventricular heterotopia	EGFR, N-cadherin	(Klezovitch et al. 2004; Beattie et al. 2017; Jossin et al. 2017)
CYFIP1	Cyfip1 KD with shRNA	Loss of AJ and apical polarity of aRGCs, caused detachment of aRGCs, neural progenitors were ectopically localized	WAVE complex components, ARP2/3	(Yoon et al. 2014)
αE-Cadherin	αE-catenin$^{fl/fl}$, Nestin-Cre	Loss of AJ and apical polarity of aRGCs, ectopic localization of neural progenitors, shortened aRG cell cycle, hedgehog signaling upregulation, increased cell survival, cortical hyperplasia	Likely hedgehog signaling effectors	(Lien et al. 2006)
E-Cadherin (Cdh1)	Cdh1 KD with DN-Cdh1	Disruption of AJ integrity, Promoted bRGC formation	NOTCH1	(Hatakeyama et al. 2014; Martinez-Martinez et al. 2016)

LGN (GPSM2)	Lgn$^{-/-}$, Lgn KD with shRNA	Altered plane of division of aRGCs, abnormal delamination of aRGCs, Increased production of BPs	Not determined	(Konno et al. 2008)
N-Cadherin	N-cad^{fl}/N-cad ^{lacZ}, D6-Cre/+	Loss of AJs of neuroepithelial cells, disorganized aRGCs and cortical laminar	Not determined	(Kadowaki et al. 2007)
αE-Catenin	Ctnna1$^{fl/fl}$, Nes-Cre, IUE	Premature delamination in VZ, Disorganized cortical tissue, increased cell cycle exit, increased neuronal differentiation, reduction in β-catenin signaling	β-catenin signaling	(Stocker and Chenn 2009)
PARD3	Pard3$^{fl/fl}$, Emx1-Cre	Increased delamination of AP, increase BP genesis	HIPPO signaling	(Liu et al. 2018)
**Transcription-related factors**				
PAX6	Pax6$^{Sey/Sey}$, Pax6$^{fl/fl}$, Emx1-Cre, Pax6 KD, Pax6 OE	Promoted non-vertical division of APs, caused acceleration of aRGC delamination, increased BP production	BAF155, BAF170, CDC42EP4, SPAG5	(Asami et al. 2011; Wong et al. 2015; Narayanan et al. 2018)
TBR2	Tbr2$^{fl/fl}$, Foxg1-Cre, Sox1-Cre	Decreased aRGC delamination and differentiation fate transition, decreased generation and proliferation of BPs, reduced neurogenesis, reduced cortical size	Not determined	(Arnold et al. 2008; Sessa et al. 2008)
	Tbr2 OE	Increased aRGC delamination, disrupted VZ integrity, caused aRGC differentiation, promoted BP fate	Not determined	(Sessa et al. 2008)

INSM1	Insm1$^{-/-}$	Reduced BP generation and neurogenesis, caused enlargement of the VZ at the expense of the SVZ, caused reduction in cortical plate thickness	NGN2	(Farkas et al. 2008)
	Insm1 OE	Increased basolateral ciliogenesis, reduced cell-cycle progression, disassembly of AJ belt, promoted aRGC delamination, increased BP generation	PLEKHA7	(Farkas et al. 2008; Wilsch-Brauninger et al. 2012; Tavano et al. 2018)
FOXN4	Foxn4 OE	Encouraged delamination and basal dispersion of Pax6-expressing aRGCs, basal migration/dispersion of TBR2-expressing BPs	Not determined	(Narayanan et al. 2018)
SCRATCH1/2	Scratch1/2 OE	Differentiation of APs to BPs or neurons, downregulation of E-cadherin, delamination of AP progenies, initiation of migration	E-cadherin	(Itoh et al. 2013a)
**Epigenetic and chromatin remodeling factors**				
BAF155	Baf155$^{fl/fl}$, Emx1-Cre, Sey/Sey, IUE	AJ dissolution, delamination of aRGCs, increased bRGC-like cell generation	Pax6, Foxn4, Cdc42ep4, Trnp1	(Narayanan et al. 2018; Kerimoglu et al. 2021)
BAF170	Baf170 OE	Increased bRGC population arising from delaminated mutant aRGC progenies	Pax6	(Narayanan et al. 2018)
BAF155 and BAF170	Baf155$^{fl/fl}$, Baf170$^{fl/fl,}$ Emx1-Cre, hGFAP-Cre	Loss of AJs, delamination of aRGCs, caused hyperproliferation of aRGCs, decreased neurogenesis, defective migration of neurons, Reduced BP genesis	WNT signaling	(Narayanan et al. 2015; Nguyen et al. 2018; Sokpor et al. 2021)

EZH2	Ezh2$^{fl/fl}$, Emx1-Cre	Delamination and depletion of aRGCs, increased BP production, reduced neurogenesis, increased astrogenesis	H3K27me3	(Pereira et al. 2010)
RBM15	Rbm15 OE	Loss of AJs, delamination of aRGCs, promoted BP (bRGC-like cell) genesis	BAF155	(Xie et al. 2019)
**Other molecular factors**				
TRNP1	Trnp1 OE	Increase in AP pool, increased BP generation	H3K9ac	(Stahl et al. 2013; Martinez-Martinez et al. 2016; Kerimoglu et al. 2021)
	Trnp1 KD	Increased BP generation	Not determined	(Stahl et al. 2013)
PDGFRβ	*Pdgfrβ* OE	Promoted BP generation neocortex	Not determined	(Lui et al. 2014)
MARCKS	Marcks$^{-/-}$	AJ disruption, delamination of aRGCs, ectopic placement of aRGCs, defective radial migration, abnormal cortical lamination	Not determined	(Weimer et al. 2009)
TAG-1	Tag-1 KD	Loss of AJ and cell polarity complexes, delamination and ectopic placement of aRGCs	Not determined	(Okamoto et al. 2013)
LGALS3BP	*Lgals3bp OE*	Downregulation of AJ, delamination of aRGCs, BP generation, led to cortical expansion and gyrification	Not determined	(Kyrousi et al. 2021)
SAS-4	Sas-4 $^{fl/fl}$, Nestin-Cre	Delamination and basal displacement of aRGCs, mitotic delay of aRGCs, upregulation of apoptosis, cortical dysplasia	Not determined	(Insolera et al. 2014)

EML1	Eml1 KD	Abnormal spindle orientations, detachment and ectopic localization of aRGCs, severe heterotopia	Not determined	(Kielar et al. 2014)
Lamin-B	$Lmnb1^{-/-}$ and $Lmnb2^{-/-}$	Abnormal spindle orientation, Led to defective neuronal migration, cortical hypoplasia,	Not determined	(Kim et al. 2011)
ID	$Id3^{-/-}$, $Id1^{fl/fl}$, $Id2^{fl/fl}$, Nestin-Cre	Caused loss of AP stemness and anchorage leading to their premature neuronal and oligodendrocyte fate commitment	Rap1GAP, RAP1	(Lyden et al. 1999; Niola et al. 2012)
USP9X	$Usp9x^{fl/fl}$, Nestin-Cre	Transient disruption of cell adhesion and polarity of APs, increased pool and ectopic localization of Tbr2+ BPs	Itch, Numb, Wnt, and Notch targets	(Premarathne et al. 2017)
LZTS1	LZTS1 OE	Promoted AP apical process retraction, induced delamination of cortical progenitor	N-Cadherin	(Kawaue et al. 2019)
	LZTS1 KD	Impaired delamination, caused radial migration retardation		
PFN1	$Pfn1^{fl/fl}$, Nes-Cre	Disruption of actin assembly in aRGCs, increased of aRGCs, increased BP production	F-Actin	(Kullmann et al. 2020)
TBC1D3	*Tbc1d3* OE	AJ disruption, delamination of neuroprogenitors, increased BP generation, including bRGCs	N-cadherin	(Ju et al. 2016; Penisson et al. 2021)
MEMO1	$Memo1^{fl/fl}$, Emx1-Cre, hGFAP-Cre	Disrupted microtubule remodeling in APs, caused AP disorganization, defective radial migration,	Not determined	(Nakagawa et al. 2019)

APC	$Apc^{fl/fl}$, Nestin-Cre, hGFAP-Cre	Loss of aRGC polarity, instability of aRGC cytoskeleton, reduced proliferation of aRGCs, impaired neurogenesis, resulted in defective neuronal migration and cortical mislamination	β-catenin	(Yokota et al. 2009)
PAFAH1B1 (LIS1)	Lis1 KD	Altered spindle orientation and mitosis in the VZ, caused N-cadherin downregulation, Blocked bRGC generation and/or amplification without affecting TBR2 generation in the presence of TBC1D3	Not determined	(Penisson et al. 2021)
AKNA	Akna OE	Loss of AJs, increased delamination of APs and the generation of BPs	Not determined	(Camargo Ortega et al. 2019)
	Akna KD	Blockage of TGFβ1-induced AJ disassembly, hampered AP delamination leading to retention of aRGCs in the VZ		
Apical radial glial cell (aRGC), basal radial glial cell (bRGC), Adherens junction (AJ), Knockdown (KD), Overexpression (OE)				
